# Supplementary material for: Structural basis of substrate recognition and membrane association by the bacterial lysyl-phosphatidylglycerol hydrolase AcvB
Source: Commun Biol. 2026 May 22;9:689. doi: 10.1038/s42003-026-10087-1 (PMC13197417; doi:10.1038/s42003-026-10087-1)
Supplement: Supplementary file 1 — Supplementary Information [file 42003_2026_10087_MOESM1_ESM.pdf]

Structural basis of substrate recognition and membrane association by the bacterial lysyl-phosphatidylglycerol hydrolase AcvB

Mizuki Hoshi[1], Daiki Matsumoto[1], and Yasunori Watanabe[2] *

[1]Graduate School of Science and Engineering, Yamagata University, 1-4-12, Kojirakawa-machi, Yamagata 990-8560, Japan

[2]Faculty of Science, Yamagata University, 1-4-12 Kojirakawa-machi, Yamagata 990-8560, Japan.

*Corresponding author: Yasunori Watanabe; Faculty of Science, Yamagata University, 1-4-12 Kojirakawa-machi, Yamagata 990-8560, Japan;
yasunori@sci.kj.yamagata-u.ac.jp; Tel. +81 23 628 4529.

Supplementary items:

Supplementary Figures 1–9

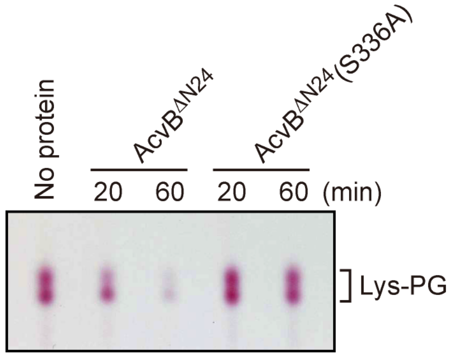

Supplementary Fig. 1 | Purified AcvB$^{\Delta N24}$ exhibits Lys-PG hydrolase activity.
Purified AcvB$^{\Delta N24}$ and the S336A variant were incubated with Lys-PG at 37 °C for the indicated times. Lipids were extracted, separated by TLC, and visualized using ninhydrin staining.

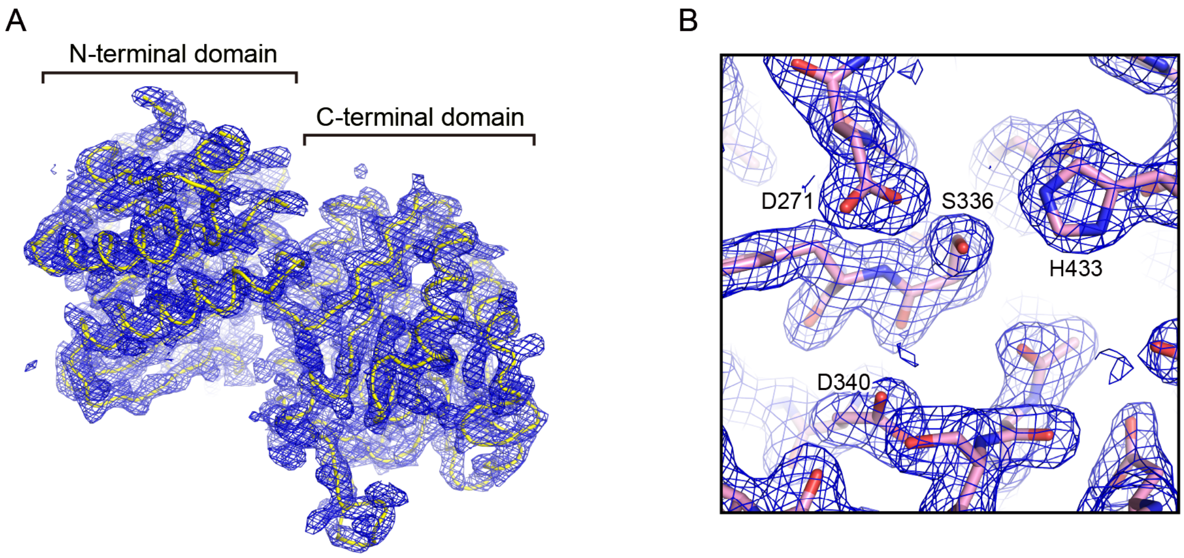

Supplementary Fig. 2 | Electron density maps of AcvB.
The 2*mF*o – *DF*c maps of (A) AcvB$^{\Delta N24}$ structure and (B) the region around the active site in the crystal structure of AcvB^{C}, shown as blue mesh and contoured at 1.0 σ.

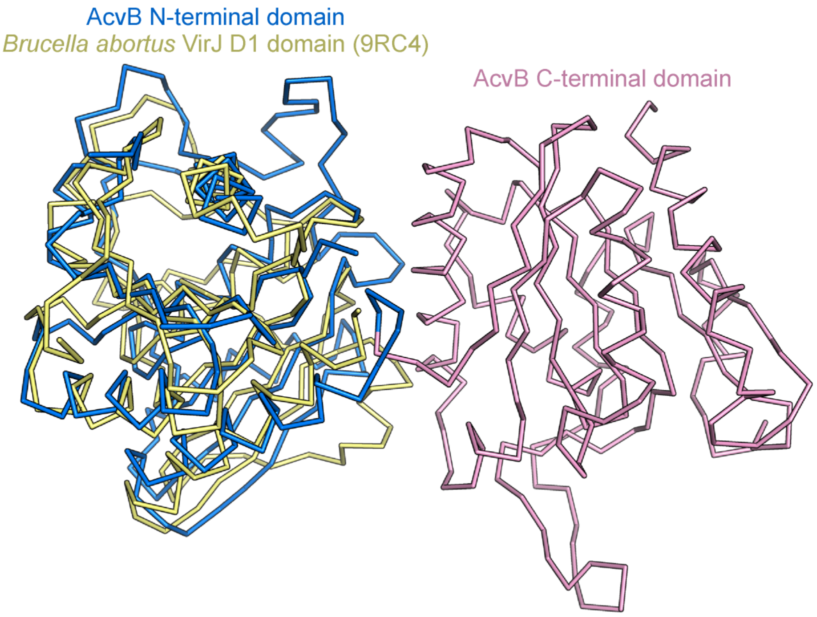

Supplementary Fig. 3 | Structural comparison of AcvB and the *B. abortus* VirJ D1 domain.

Superposition of AcvB$^{\Delta N24}$ and the *B. abortus* VirJ D1 domain (PDB code 9RC4). The N-terminal and C-terminal domains of AcvB$^{\Delta N24}$ are colored blue and pink, respectively, whereas the *B. abortus* VirJ D1 domain is colored red and yellow.

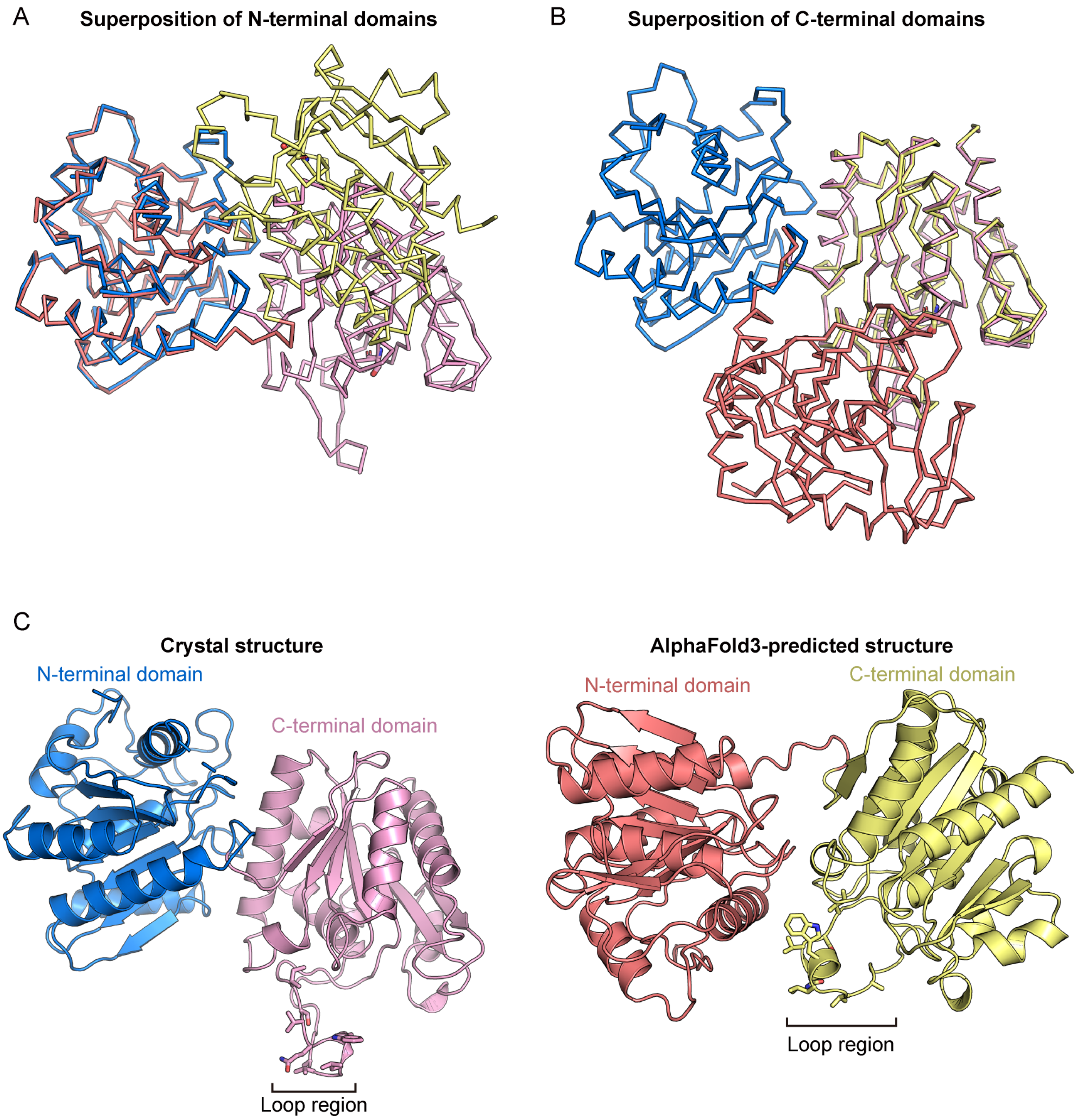

Supplementary Fig. 4 | Comparison of the crystal and AlphaFold3-predicted structures of AcvB$^{\Delta N24}$.

(A) Superposition of the N-terminal domains of AcvB$^{\Delta N24}$ in the crystal structure and the model predicted by AlphaFold3. The N-terminal and C-terminal domains of the crystal structure are colored blue and pink, respectively, whereas those of the AlphaFold3-predicted model are colored red and yellow, respectively. (B) Superposition of the C-terminal domains of AcvB$^{\Delta N24}$ in the crystal structure and AlphaFold3-predicted model. (C) Crystal structure (left) and AlphaFold3-predicted structure (right). The hydrophobic protruding loop regions are indicated.

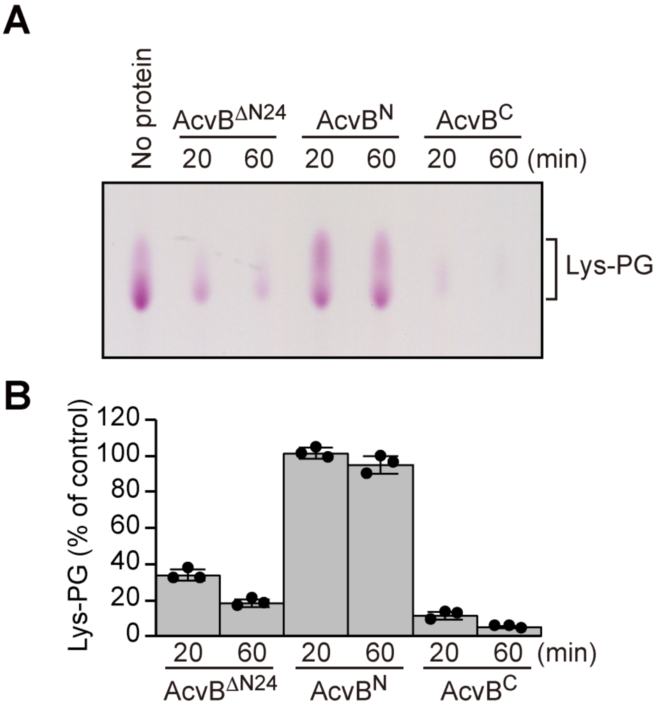

Supplementary Fig. 5 | Lys-PG hydrolase activities of the N- and C-terminal domains of AcvB.

(A) Purified AcvB$^{\Delta N24}$, AcvB^{N}, and AcvB^{C} were incubated with Lys-PG at 37 °C for the indicated times. Lipids were extracted, separated by TLC, and visualized using ninhydrin staining. (B) Quantification of Lys-PG based on the data in (A), using ImageJ. Values are expressed relative to the no-protein control and represent mean ± SD from three independent experiments (n = 3).

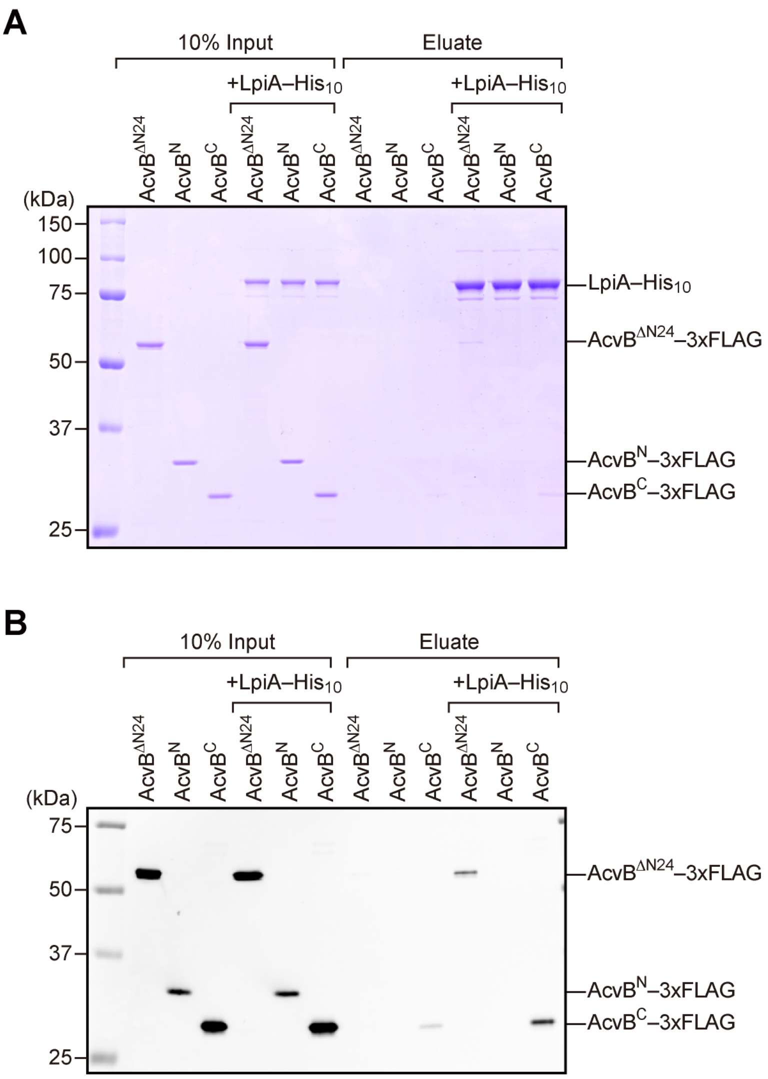

Supplementary Fig. 6 | In vitro Ni-NTA pull-down assay showing the interaction between AcvB and LpiA.

C-terminally His_{10}-tagged LpiA (LpiA–His_{10}) bound to Ni-NTA resin was incubated separately with C-terminally 3xFLAG-tagged AcvB variants ($AcvB^{ΔN24}$, $AcvB^{N}$, and $AcvB^{C}$). Proteins bound to the resin were eluted and analyzed using SDS-PAGE followed by CBB staining (A) and immunoblotting with anti-FLAG antibody (B).

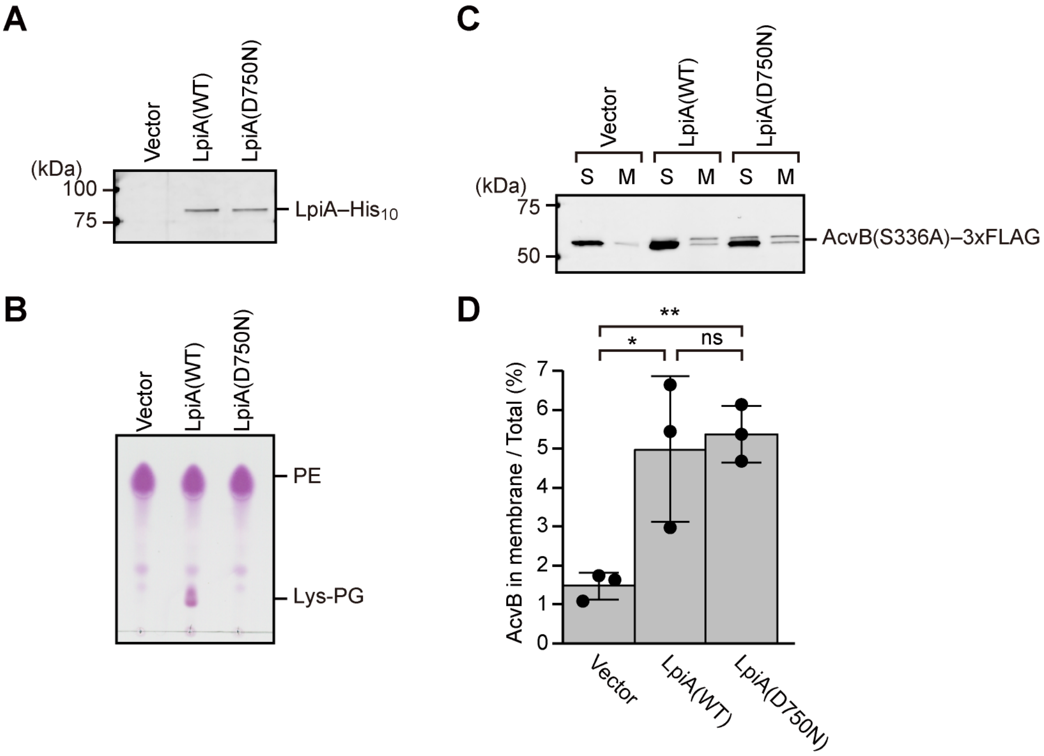

Supplementary Fig. 7 | LpiA enhances AcvB membrane association.
(A) *E. coli* cells harboring expression plasmids encoding C-terminally His_{10}-tagged LpiA (LpiA–His_{10}) variants and the C-terminally 3xFLAG-tagged AcvB S336A variant (AcvB(S336A)–3xFLAG) were cultured at 37 °C. When the OD_{600} reached ~0.5, IPTG was added to a final concentration of 0.5 mM and the cells were incubated at 37 °C for 3 h. Cell lysates were subjected to SDS-PAGE, followed by immunoblotting with anti-6x His antibody to detect LpiA. (B) Total phospholipids were extracted from *E. coli* cells expressing LpiA–His_{10} variants and AcvB(S336A)–3xFLAG, separated by TLC, and visualized using ninhydrin staining. (C) Soluble (S) and membrane (M) fractions were separated from *E. coli* cells expressing LpiA-His_{10} variants and AcvB(S336A)–3xFLAG and subjected to SDS-PAGE and immunoblotting with anti-6x His antibody to detect AcvB. (D) Quantification of AcvB in the membrane fraction based on the data in (C), using ImageJ. Values represent mean ± SD from three independent experiments (n = 3). *, $p < 0.05$; **, $p < 0.01$, p-values were obtained from the unpaired two-tailed t-test. ns, not significant.

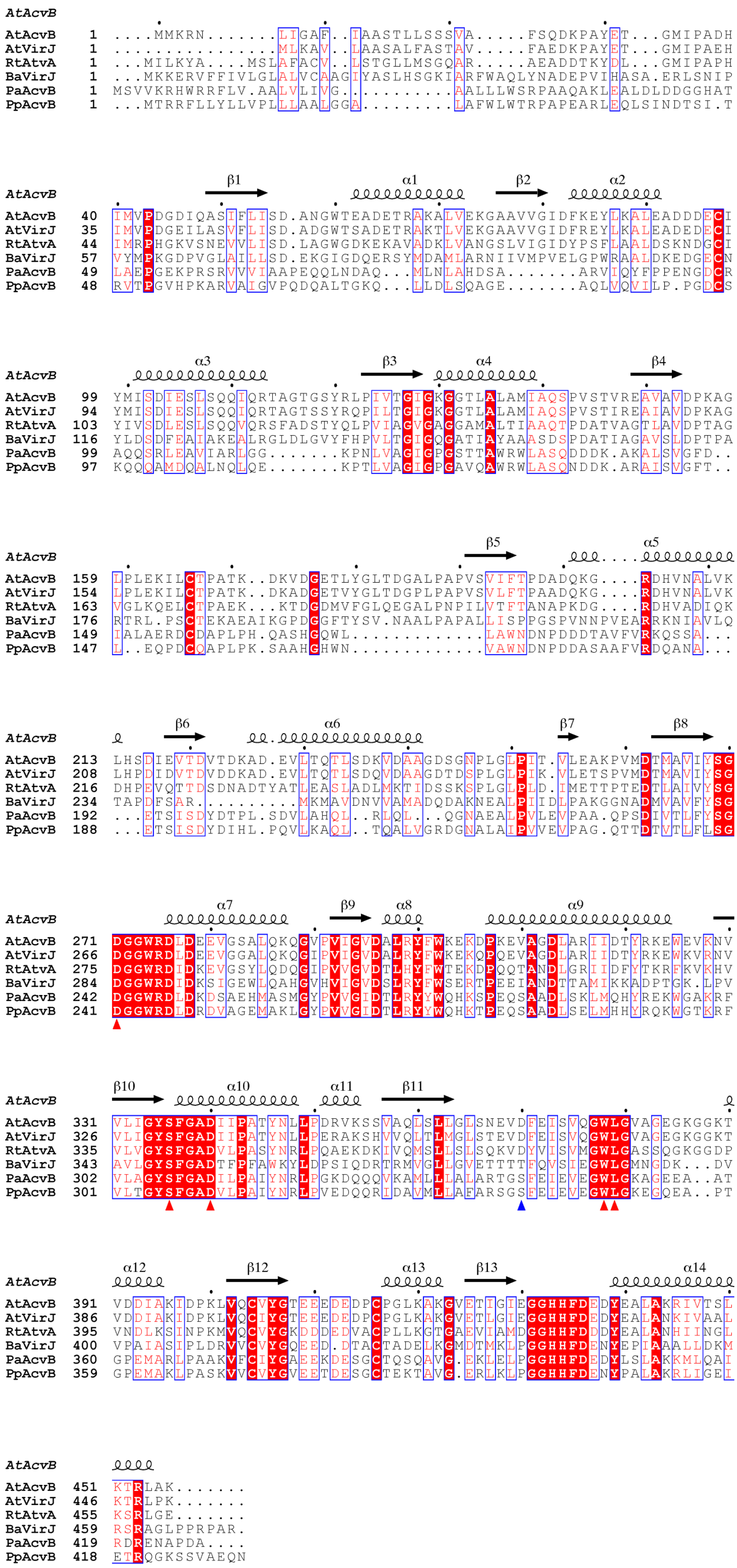
AtAcvB
AtAcvB 1 ....MMKRN.......LIGAF..IAASTLLSSSVA.....FSQDKPAYET...GMIPADH
AtVirJ 1 ...............MLKAV..LAASALFASTAV.....FAEDKPAYET...GMIPAEH
RtAtvA 1 ...MILKYA....MSLAFACV..LSTGLLMSGQAR.....AEADDTKYDL...GMIPAPH
BaVirJ 1 ...MKKERVFFIVLGLALVCAAGIYASLHSGKIARFWAQLYNADEPVIHASA.ERLSNIP
PaAcvB 1 MSVVKRHWRRFLV.AALVLIVG...........AALLLWSRPAAQAKLEALDLDDGGHAT
PpAcvB 1 ...MTRRFLLYLLVPLLLAALGGA.........LAFWLWTRPAPEARLEQLSINDTSI.T
β1 α1 β2 α2
AtAcvB
AtAcvB 40 IMVPDGDIQASIFLISD.ANGWTEADETRAKALVEKGAAVVGIDFKEYLKALEADDDECI
AtVirJ 35 IMVPDGEILASVFLISD.ADGWTSADETRAKTLVEKGAAVVGIDFREYLKALEADEDECI
RtAtvA 44 IMRPHGKVSNEVVLISD.LAGWGDKEKAVADKLVANGSLVIGIDYPSFLAALDSKNDGCI
BaVirJ 57 VYMPKGDPVGLAILLSD.EKGIGDQERSYMDAMLARNIIVMPVELGPWRAALDKEDGECN
PaAcvB 49 LAEPGEKPRSRVVVIAAPEQQLNDAQ...MLNLAHDSA.......ARVIQYFPPENGDCR
PpAcvB 48 RVTPGVHPKARVAIGVPQDQALTGKQ...LLDLSQAGE.......AQLVQVILP.PGDCS
α3 β3 α4 β4
AtAcvB
AtAcvB 99 YMISDIESLSQQIQRTAGTGSYRLPIVTGIGKGGTLALAMIAQSPVSTVREAVAVDPKAG
AtVirJ 94 YMISDIESLSQQIQRTAGTSSYRQPILTGIGKGGTLALAMIAQSPVSTIREAIAVDPKAG
RtAtvA 103 YIVSDLESLSQQVQRSFADSTYQLPVIAGVGAGGAMALTIAAQTPDATVAGTLAVDPTAG
BaVirJ 116 YLDSDFEAIAKEALRGLDLGVYFHPVLTGIGQGATIAYAAASDSPDATIAGAVSLDPTPA
PaAcvB 99 AQQSRLEAVIARLGG.......KPNLVAGIGPGSTTAWRWLASQDDDK.AKALSVGFD..
PpAcvB 97 KQQQAMDQALNQLQE.......KPTLVAGIGPGAVQAWRWLASQNDDK.ARAISVGFT..
β5 α5
AtAcvB
AtAcvB 159 LPLEKILCTPATK..DKVDGETLYGLTDGALPAPVSVIFTPDADQKG....RDHVNALVK
AtVirJ 154 LPLEKILCTPATK..DKADGETVYGLTDGPLPAPVSVLFTPAADQKG....RDHVNALVK
RtAtvA 163 VGLKQELCTPAEK...KTDGDMVFGLQEGALPNPILVTFTANAPKDG....RDHVADIQK
BaVirJ 176 RTRL.PSCTEKAEAIKGPDGGFTYSV.NAALPAPALLISPPGSPVNNPVEARRKNIAVLQ
PaAcvB 149 IALAERDCDAPLPH.QASHGQWL.............LAWNDNPDDDTAVFVRKQSSA...
PpAcvB 147 L..EQPDCQAPLPK.SAAHGHWN.............VAWNDNPDDASAAFVRDQANA...
β6 α6 β7 β8
AtAcvB
AtAcvB 213 LHSDIEVTDVTDKAD.EVLTQTLSDKVDAAGDSGNPLGLPIT.VLEAKPVMDTMAVIYSG
AtVirJ 208 LHPDIDVTDVDDKAD.EVLTQTLSDQVDAAGDTDSPLGLPIK.VLETSPVMDTMAVIYSG
RtAtvA 216 DHPEVQTTDSDNADTYATLEASLADLMKTIDSSKSPLGLPLD.IMETTPTEDTLAIVYSG
BaVirJ 234 TAPDFSAR.........MKMAVDNVVAMADQDAKNEALPIIDLPAKGGNADMVAVFYSG
PaAcvB 192 ...ETSISDYDTPL.SDVLAHQL..RLQL...QGNAEALPVLEVPAA.QPSDIVTLFYSG
PpAcvB 188 ...ETSISDYDIHL.PQVLKAQL..TQALVGRDGNALAIPVVEVPAG.QTTDTVTLFLSG
α7 β9 α8 α9
AtAcvB
AtAcvB 271 DGGWRDLDEEVGSALQKQGVPVIGVDALRYFWKEKDPKEVAGDLARIIDTYRKEWEVKNV
AtVirJ 266 DGGWRDLDEEVGGALQKQGIPVIGVDALRYFWKEKQPQEVAGDLARIIDTYRKEWKVRNV
RtAtvA 275 DGGWRDIDKEVGSYLQDQGIPVVGVDTLHYFWTEKDPQQTANDLGRIIDFYTKRFKVKHV
BaVirJ 284 DGGWRDIDKSIGEWLQAHGVHVIGVDSLRYFWSERTPEEIANDTTAMIKKADPTGK.LPV
PaAcvB 242 DGGWRDLDKDSAEHMASMGYPVVGIDTLRYYWQHKSPEQSAADLSKLMQHYREKWGAKRF
PpAcvB 241 DGGWRDLDRDVAGEMAKLGYPVVGIDTLRYYWQHKTPEQSAADLSELMHHYRQKWGTKRF
β10 α10 α11 β11
AtAcvB
AtAcvB 331 VLIGYSFGADIIPATYNLLPDRVKSSVAQLSLLGLSNEVDFEISVQGWLGVAGEGKGGKT
AtVirJ 326 VLIGYSFGADIIPATYNLLPERAKSHVVQLTLMGLSTEVDFEISVQGWLGVAGEGKGGKT
RtAtvA 335 VLVGYSFGADVLPASYNRLPQAEKDKIVQMSLLSLSQKVDYVISVMGWLGASSQGKGGDP
BaVirJ 343 AVLGYSFGADTFPFAWKYLDPSIQDRTRMVGLLGVETTTTFQVSIEGWLGMNGDK...DV
PaAcvB 302 VLAGYSFGADILPAIYNRLPGKDQQQVKAMLLLALARTGSFEIEVEGWLGKAGEEA..AT
PpAcvB 301 VLTGYSFGADVLPAIYNRLPVEDQQRIDAVMLLAFARSGSFEIEVEGWLGKEGQEA..PT
α12 β12 α13 β13 α14
AtAcvB
AtAcvB 391 VDDIAKIDPKLVQCVYGTEEEDEDPCPGLKAKGVETIGIEGGHHFDEDYEALAKRIVTSL
AtVirJ 386 VDDIAKIDPKLVQCIYGTEEEDEDPCPGLKAKGVETLGIEGGHHFDEDYEALANKIVAAL
RtAtvA 395 VNDLKSINPKMVQCVYGKDDDEDVACPLLKGTGAEVIAMDGGHHFDDDYEALANHIINGL
BaVirJ 400 VPAIASIPLDRVVCVYGQEED.DTACTADELKGMDTMKLPGGHHFDENYEPIAAALLDKM
PaAcvB 360 GPEMARLPAAKVFCIYGAEEKDESGCTQSQAVG.EKLELPGGHHFDEDYLSLAKKMLQAI
PpAcvB 359 GPEMAKLPASKVVCVYGVEETDESGCTEKTAVG.ERLKLPGGHHFDENYPALAKRLIGEI
AtAcvB
AtAcvB 451 KTRLAK.......
AtVirJ 446 KTRLPK.......
RtAtvA 455 KSRLGE.......
BaVirJ 459 RSRAGLPPRPAR.
PaAcvB 419 RDRENAPDA....
PpAcvB 418 ETRQGKSSVAEQN

Supplementary Fig. 8 | Sequence alignment of AcvB homologs.

Multiple-sequence alignment of *Agrobacterium tumefaciens* AcvB (AtAcvB) and its homologs: *A. tumefaciens* VirJ (AtVirJ), *Rhizobium tropici* AtvA (RtAtvA), *Brucella abortus* VirJ (BaVirJ), PA0919 from *Pseudomonas aeruginosa* (PaAcvB), and PP_1201 from *Pseudomonas putida* (PpAcvB). Identical residues are shaded red, similar residues are colored red, and similar residues across groups are boxed in blue. The secondary structure of AcvB is shown above the sequences. Conserved residues Asp271, Ser336, Asp340, Trp378, and Leu379 in AcvB are indicated by red arrowheads, and Asp370 is indicated by a blue arrowhead.

Figure 2C upper panel

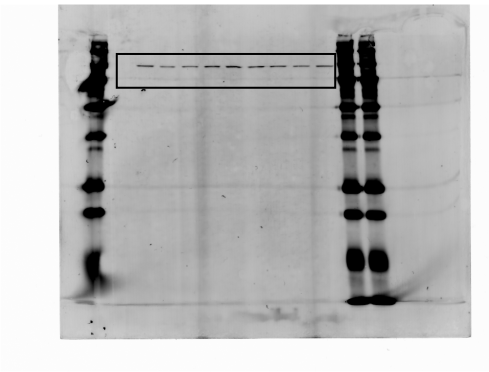

Figure 2D

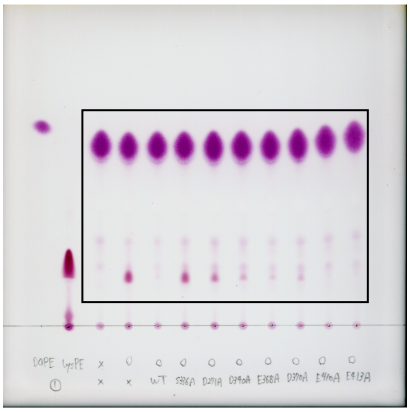

Figure 3C Figure 5F

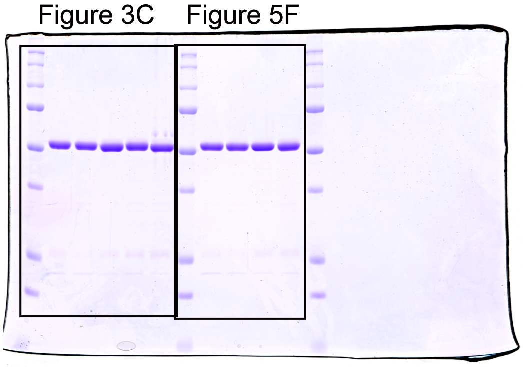

Figure 2C lower panel

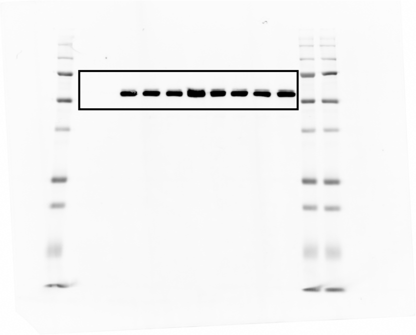

Figure 3D

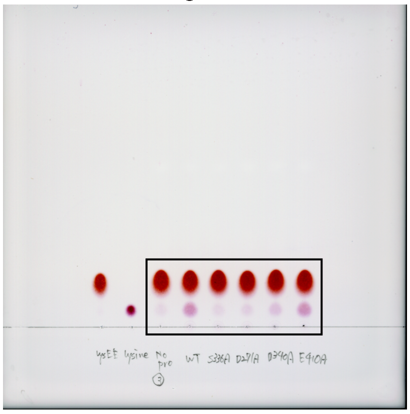

Figure 5G

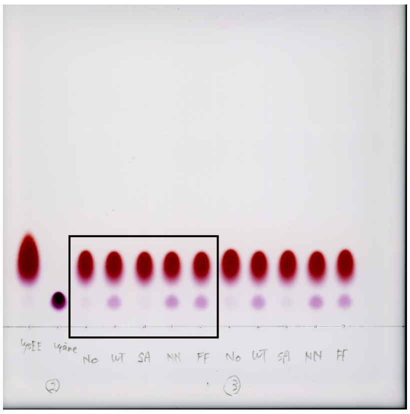

Figure 5B top panel

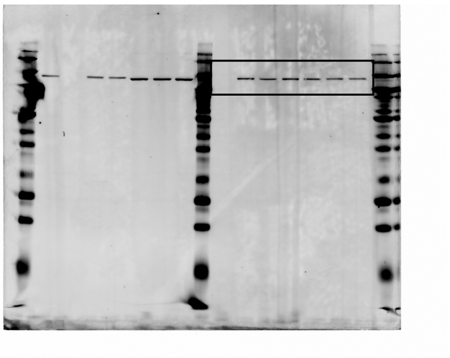

Figure 5D

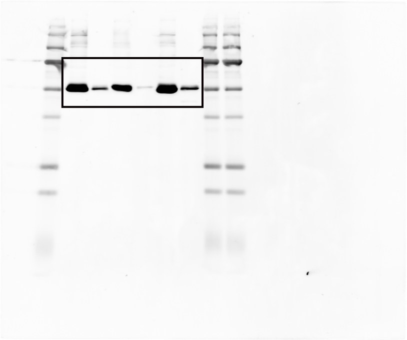

Figure 6A upper panel

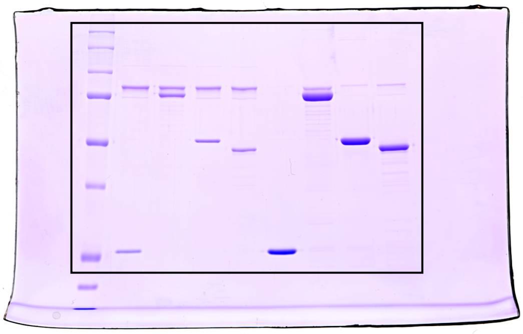

Figure 5B middle panel

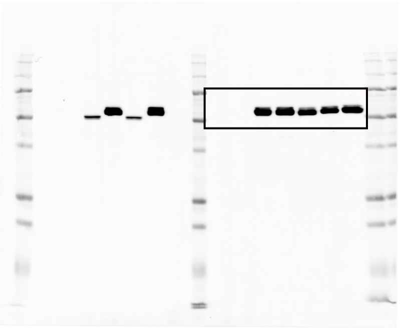

Figure 5B bottom panel

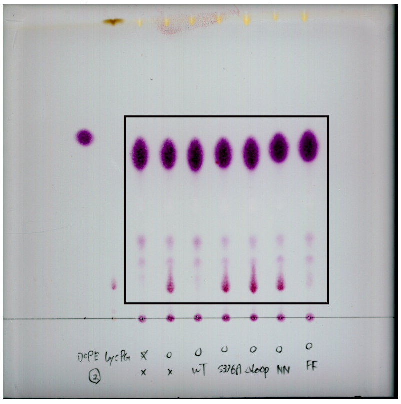

Figure 6A lower panel

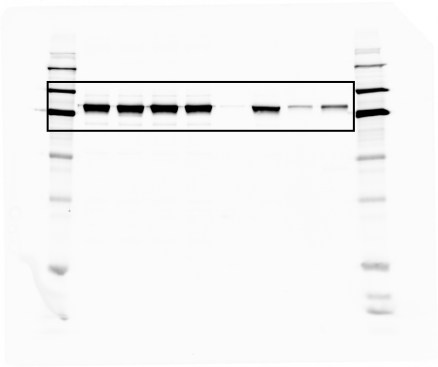

Supplementary Figure 1

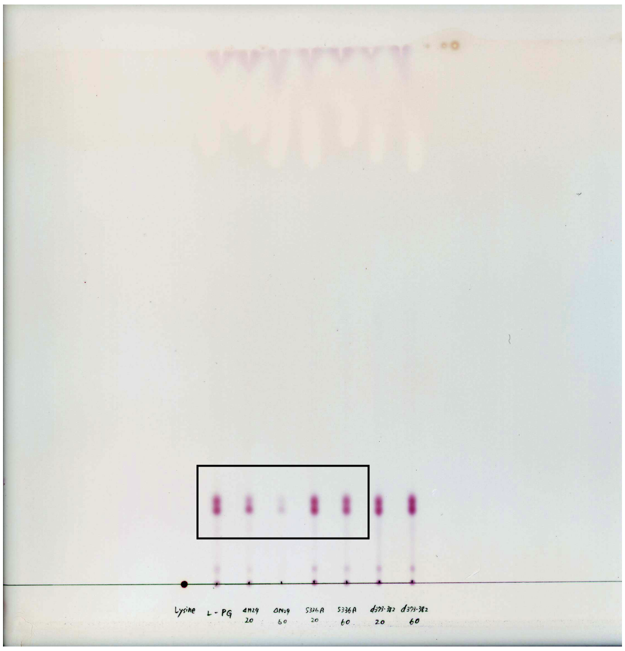

Supplementary Figure 5A

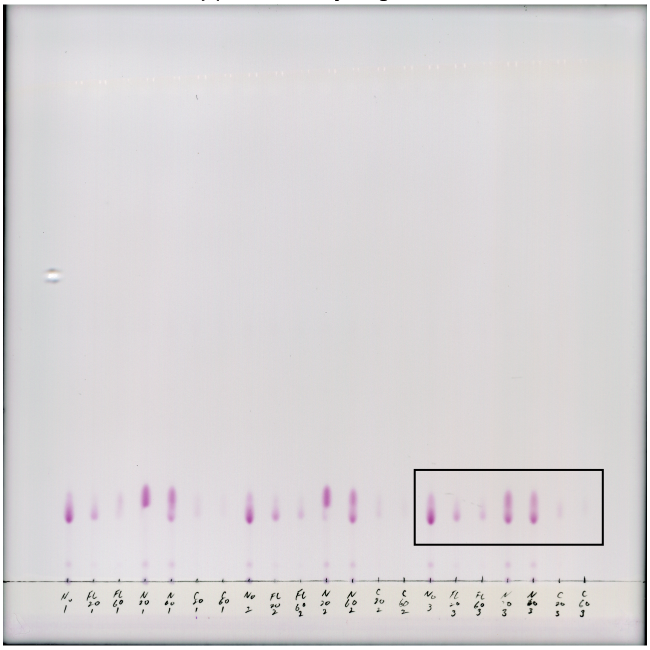

Supplementary Figure 6A

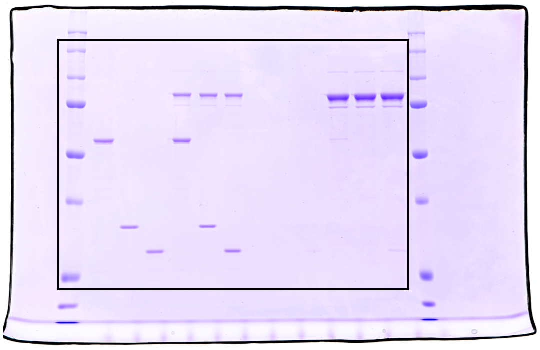

Supplementary Figure 7A

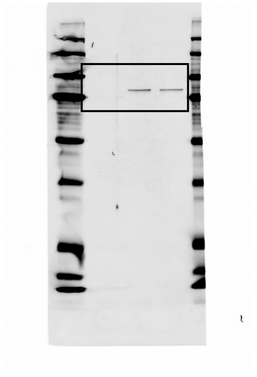

Supplementary Figure 7B

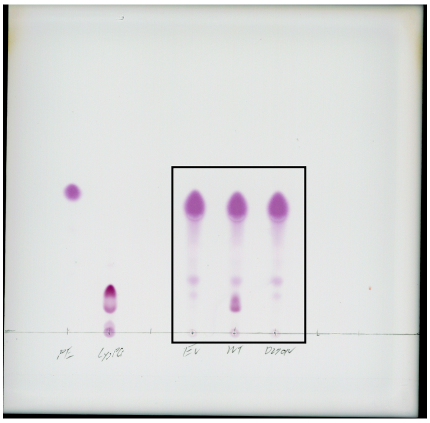

Supplementary Figure 6B

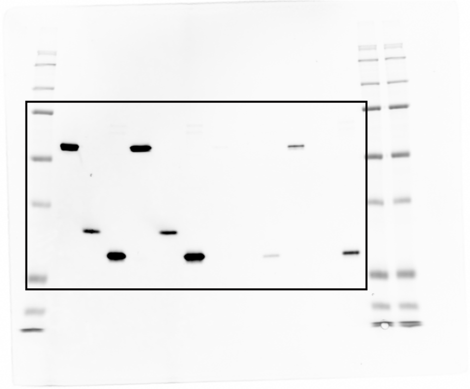

Supplementary Figure 7C

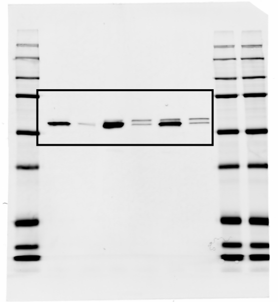

Supplementary Fig. 9 | Uncropped images of blots, gels, and TLC plates.
Boxes mark the borders of the final cropped images.
