# Supplementary material for: Structural basis of substrate recognition and membrane association by the bacterial lysyl-phosphatidylglycerol hydrolase AcvB
Source: Commun Biol. 2026 May 22;9:689. doi: 10.1038/s42003-026-10087-1 (PMC13197417; doi:10.1038/s42003-026-10087-1)
Supplement: Supplementary file 2 — Description of Additional Supplementary Files [file 42003_2026_10087_MOESM2_ESM.docx]

**Description of Additional Supplementary File**

File name : Supplementary data 1
Description: Source data for graphs are available in Supplementary Data 1.
