# Supplementary material for: Structural basis of substrate recognition and membrane association by the bacterial lysyl-phosphatidylglycerol hydrolase AcvB
Source: Commun Biol. 2026 May 22;9:689. doi: 10.1038/s42003-026-10087-1 (PMC13197417; doi:10.1038/s42003-026-10087-1)
Supplement: Supplementary file 4 — Reporting Summary [file 42003_2026_10087_MOESM4_ESM.pdf]

nature portfolio

Corresponding author(s): Yasunori Watanabe

Last updated by author(s): Mar 31, 2026

Reporting Summary

Nature Portfolio wishes to improve the reproducibility of the work that we publish. This form provides structure for consistency and transparency in reporting. For further information on Nature Portfolio policies, see our Editorial Policies and the Editorial Policy Checklist.

Statistics

For all statistical analyses, confirm that the following items are present in the figure legend, table legend, main text, or Methods section.

n/a	Confirmed	
☐	☒	The exact sample size (*n*) for each experimental group/condition, given as a discrete number and unit of measurement
☐	☒	A statement on whether measurements were taken from distinct samples or whether the same sample was measured repeatedly
☐	☒	The statistical test(s) used AND whether they are one- or two-sided<br>*Only common tests should be described solely by name; describe more complex techniques in the Methods section.*
☒	☐	A description of all covariates tested
☒	☐	A description of any assumptions or corrections, such as tests of normality and adjustment for multiple comparisons
☐	☒	A full description of the statistical parameters including central tendency (e.g. means) or other basic estimates (e.g. regression coefficient) AND variation (e.g. standard deviation) or associated estimates of uncertainty (e.g. confidence intervals)
☒	☐	For null hypothesis testing, the test statistic (e.g. *F*, *t*, *r*) with confidence intervals, effect sizes, degrees of freedom and *P* value noted<br>*Give P values as exact values whenever suitable.*
☒	☐	For Bayesian analysis, information on the choice of priors and Markov chain Monte Carlo settings
☒	☐	For hierarchical and complex designs, identification of the appropriate level for tests and full reporting of outcomes
☒	☐	Estimates of effect sizes (e.g. Cohen's *d*, Pearson's *r*), indicating how they were calculated

Our web collection on statistics for biologists contains articles on many of the points above.

Software and code

Policy information about availability of computer code

Data collection	X-ray diffraction data were collected at the SPring-8 beamline BL32XU using an EIGER X 9M detector (DECTRIS). The diffraction data were processed using the KAMO and XDS software. Fluorescent immunoblot signals were detected using a Typhoon FLA 9500 imaging system (GE Healthcare).
Data analysis	ImageJ software was used for quantification of immunoblot and TLC plate signals. Molecular replacement was performed using PHASER. Model building was carried out using COOT. Crystallographic refinement was performed using PHENIX. Assessment of the quality and geometry of structural models was performed using MolProbity. Molecular docking was performed using the SwissDock web server (based on AutoDock Vina). Protein sequence alignment analysis was performed using Clustal Omega and ESPript 3.0. All structural figures were prepared with PyMOL.

For manuscripts utilizing custom algorithms or software that are central to the research but not yet described in published literature, software must be made available to editors and reviewers. We strongly encourage code deposition in a community repository (e.g. GitHub). See the Nature Portfolio guidelines for submitting code & software for further information.

Data

Policy information about availability of data

All manuscripts must include a data availability statement. This statement should provide the following information, where applicable:

- Accession codes, unique identifiers, or web links for publicly available datasets
- A description of any restrictions on data availability
- For clinical datasets or third party data, please ensure that the statement adheres to our policy

The atomic coordinates and structure factor files have been deposited in the Protein Data Bank under accession codes 9XHM (AcvB(25–456)) and 9XHN (AcvB(249–456)). All other data are available within the article and Supplementary Information or from the corresponding author upon request.

Research involving human participants, their data, or biological material

Policy information about studies with human participants or human data. See also policy information about sex, gender (identity/presentation), and sexual orientation and race, ethnicity and racism.

Reporting on sex and gender	n/a
Reporting on race, ethnicity, or other socially relevant groupings	n/a
Population characteristics	n/a
Recruitment	n/a
Ethics oversight	n/a

Note that full information on the approval of the study protocol must also be provided in the manuscript.

Field-specific reporting

Please select the one below that is the best fit for your research. If you are not sure, read the appropriate sections before making your selection.

☒ Life sciences ☐ Behavioural & social sciences ☐ Ecological, evolutionary & environmental sciences

For a reference copy of the document with all sections, see nature.com/documents/nr-reporting-summary-flat.pdf

Life sciences study design

All studies must disclose on these points even when the disclosure is negative.

Sample size	Biochemical assays (Lys-PG and Lys-EE hydrolase activity and membrane association assays) were conducted using three independent biological replicates, which provided reproducible results.
Data exclusions	No data were excluded from the analyses.
Replication	All replicate experiments were successfully reproduced.
Randomization	n/a
Blinding	n/a

Reporting for specific materials, systems and methods

We require information from authors about some types of materials, experimental systems and methods used in many studies. Here, indicate whether each material, system or method listed is relevant to your study. If you are not sure if a list item applies to your research, read the appropriate section before selecting a response.

Materials & experimental systems

n/a	Involved in the study
☐	☒ Antibodies
☒	☐ Eukaryotic cell lines
☒	☐ Palaeontology and archaeology
☒	☐ Animals and other organisms
☒	☐ Clinical data
☒	☐ Dual use research of concern
☒	☐ Plants

Methods

n/a	Involved in the study
☒	☐ ChIP-seq
☒	☐ Flow cytometry
☒	☐ MRI-based neuroimaging

Antibodies

Antibodies used

Anti-6x His antibody (FUJIFILM Wako, Cat. # 011-23091)
Anti-FLAG antibody (Sigma-Aldrich, Cat. # F1804)
Goat anti-mouse IgG (H + L) cross-absorbed secondary antibody, cyanine5 (Life Technologies, Cat. # A10524)

Validation

All primary and secondary antibodies used in this study are commercially available and widely validated for Western blotting. Anti-6x His and anti-FLAG antibodies gave single bands at expected molecular weights. The secondary antibody generated signals only in the presence of primary antibodies.

Plants

Seed stocks

n/a

Novel plant genotypes

n/a

Authentication

n/a
